# Meta-Analysis of miRNAs and Their Involvement as Biomarkers in Oral Cancers

**DOI:** 10.1155/2018/8439820

**Published:** 2018-01-04

**Authors:** Andleeb Zahra, Itrat Rubab, Sumaira Malik, Amina Khan, Muhammad Jawad Khan, M. Qaiser Fatmi

**Affiliations:** COMSATS Institute of Information Technology, Park Road, Chak Shahzad, Islamabad 45600, Pakistan

## Abstract

Oral Squamous Cell Carcinoma (OSCC) is one of the most common cancers worldwide. Recent studies have highlighted the role of miRNA in disease pathology, indicating its potential use as an early diagnostic marker. Dysregulated expression of miRNAs is known to affect cell growth, and these may function as tumor suppressors or oncogenes in various cancers. The main objective of this study was to characterize the extracellular miRNAs involved in* oral cancer* (OC) that can potentially be used as biomarkers of OC. A total of 318 miRNAs involved in oral carcinoma were shortlisted.* Differentially expressed genes* (DEGs) of oral carcinoma from reported experiments were identified. Common genes between lists of DEGs of OC of each miRNA were identified. These common genes are the targets of specific miRNA, which may be used as biomarkers of OC. A list of significant biomarkers for cancer was generated like CDH2 and CDK7, and functional enrichment analysis identified the role of miRNAs in major pathways like cell adhesion molecules pathway affected by cancer. We observed that at least 25 genes like* ABCF3*,* ALDH2*,* CD163L1*, and so forth are regulated by a maximum number of miRNAs; thereby, they can be used as biomarkers of OC.

## 1. Introduction

Oral Squamous Cell Carcinoma (OSCC) has become a dreadful health issue annually affecting approximately 481,000 new patients worldwide [1]. It is ranked as sixth most common cancer worldwide [2]. In Europe, smoking and alcohol drinking while in Asia, betel quid, tobacco, bidi or gutka, low quality food colours, and human papillomavirus infections are chief fabricators of OSCC. [3–5]. Despite the advent of new technology, yet there is a lack of comprehensive screening procedure for this disease. It has been reported that the patient's survival rate will approach 80% if the OC has been diagnosed at initial stages, that is, stages 0 to 2, and survival rate decreases to 20–40% if the OC is diagnosed at final stages, that is, stage 4 or later [6]. Like all major types of cancers, for example, breast cancer and lung cancer, the OC also needs a distinctive marker which can be utilized to detect the disease at the earliest possible stages. Multiple efforts have been already made in this context [2, 7, 8], and particularly miRNAs have been greatly focused during the last decade hoping them to be potential biomarkers due to their reported role in OC [2, 9, 10]. The miRNA, involved in gene regulation by inhibition or stimulation, is composed of 18 to 24 evolutionarily conserved nucleotides [2, 11]. Different human cancers like lung cancer, acute lymphoblastic leukaemia, and colon cancer are caused by the upregulation of oncogenes and downregulation of tumor suppressors due to the binding of miRNA at 30 untranslated regions (30 UTR) [12, 13]. The extracellular and tumor miRNAs are significantly relevant to cancers due to their presence in different body fluids [14]. These cell-free miRNAs are dramatically powerful biomarkers for OC due to their extraordinary features including stability, conservation, and discriminatory power. Turchinovich et al. [15] have shown that miRNA can remain stable in the extracellular space as a by-product for at least 1 month showing the stability of miRNAs and making them ideal candidates for biomarkers. It has been reported that differential expression of miRNA is observed between healthy and oral cancerous persons [16]. Validation of multiple miRNA expression levels can differentiate cancer types. This phenomenon is named as miRNA signatures [14]. Further study has revealed a high level of specificity of miRNA at tissue and cancer stages of OSCC [12]. Ultimately, miRNAs have all mandatory features to be an early diagnostic marker.

Contrary to miRNAs present in other body fluids, salivary miRNAs are considered more important in OC. Some salivary miRNAs like miR-200a and miR-125a are very sensitive and their concentration significantly decreases in OC patients; therefore, they can be used as markers for comparison of healthy and diseased persons [16]. Being an easily accessible body fluid, saliva has been used for diagnostic purposes for many decades. Saliva is a reliable diagnostic fluid in various types of cancers including squamous cell carcinoma of oral cavity, where both specific and nonspecific tumor markers are used [17]. miRNAs are present in both whole saliva and supernatant saliva. Two of these miRNAs, that is, miR-125a and miR-200a, are differentially expressed in the saliva of the OC patients compared with those of healthy controls [12].

The main objective of this in silico study is to find a set of salivary miRNAs and their differentially expressed target genes. These sets of genes and miRNAs have discriminatory power for detection of OC and may lead towards the discovery of discrete biomarkers for OC.

## 2. Materials and Methods

For in silico studies, the research data published during 2009–2014 were collected using different keywords or their combinations like “Expression profiling by array”, “Oral cancers”, “human oral carcinoma”, “gene expression profiling of human oral cancers”, and so forth. Experiments were selected on the basis of differential expression of genes study related to OC with good sample size. Selected experiments are shown in Supplementary Table 1. Furthermore, miRNAs involved in OC were selected based on the following criteria: (1) cell-free salivary miRNA, and (2) involvement in oral cancer. Secondary structures of these miRNAs were taken from miRNAMap. Expression of genes involved in oral cancer from different types of experiments was analyzed by geo2R (http://www.ncbi.nlm.nih.gov/geo/). After the analysis, the gene expression data of each experiment was obtained in the form of Microsoft Excel files, containing gene symbols, *p* values, and gene log fold change (FC) values. Only genes with *p* value less than 0.05 were selected for further analysis.

DIANA-Micro T-CDS tool [19] was used to identify target genes for each miRNA. This web server tool predicts and analyzes targets of miRNA. Target prediction algorithm is used at the backend of this server. As an input, it takes the name of miRNA and generates a file enlisting all those target genes with their ensemble IDs, which are regulated by that specific miRNA. After obtaining all DEGs in OC as well as target genes of all miRNAs in two separate files, the common genes in both files were extracted and compared with target genes. Gene symbols in both files were compared, and gene symbols along with ensemble ID and log FC of matched genes were retrieved against miRNA. This strategy was used to retrieve common genes which are summarized in Algorithm 1 in Supplementary File 1.

**Figure 1:**
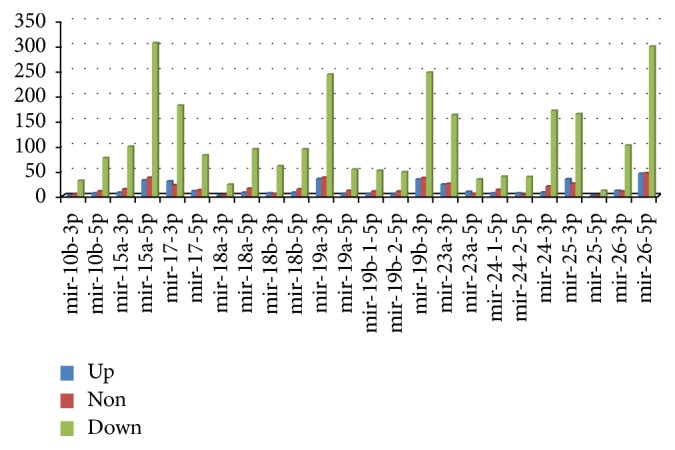
Number of genes regulated against miRNA. Blue, red, and green bars are showing the upregulation, nonregulation, and downregulation of genes, respectively. The miRNAs are shown on the *x*-axis of the graph while genes are represented on the *y*-axis.

After retrieving common genes from DEGs in OC and target genes of each of miRNA, a set of genes was generated being regulated by a maximum number of miRNAs (involved in OC) [20]. Common genes from IPA and our data were analyzed through the use of QIAGEN's Ingenuity Pathway Analysis (IPA®, QIAGEN Redwood City, https://www.qiagen.com/ingenuity). A list of gene names was provided as an input to IPA, and as an output, it provided genes with their gene name, site information, and type. Pathways and biomarker studies were also performed using IPA. After selecting differentially expressed genes in cancer as well as the targets of miRNA, genes that were upregulated, downregulated, and nonregulated were identified for each miRNA using Kyoto Encyclopedia of Genes and Genomes (KEGG). The results are shown in Figure 1 and Supplementary Figure 1(b–l). These results were obtained by using KEGG array. The results show that most of the genes are downregulated.

We also performed the functional analysis of DEGs through an online tool Database for Annotation, Visualization and Integrated Discover (DAVID). Here, we present data of 5 miRNAs and their target DEGs. The DEGs for each of miRNA were separately submitted to the functional annotation utility provided by DAVID. The background was selected to be* Homo sapiens*. DAVID associates each gene identifier with a GO term [21]. This GO term is actually the name of the functional characteristic of that particular gene. We included “biological process,” “molecular function,” and “cellular compartments” categories of DAVID functional tool.

## 3. Results and Discussion

Total number of DEGs from all experiments and specific techniques used to measure gene expression are shown in Table 1.

A total of 318 miRNAs were found to be involved in OC, which are regulating around ≈16000 genes. These genes are validated DEGs in oral cancer. After retrieving this information, the expression pattern (either upregulation or downregulation) of genes, that is, the target of each miRNA, was measured by Kyoto Encyclopedia of Genes and Genomes (KEGG). The results of 24 miRNAs are shown in Figure 1(a) and all other miRNAs can be seen in Supplementary Figure 1(b–l).

From Ingenuity knowledge base, a list of unspecified biomarkers of oral cancer is presented in Supplementary Table 2 along with the number of miRNAs and their target specific gene.

**Figure 2:**
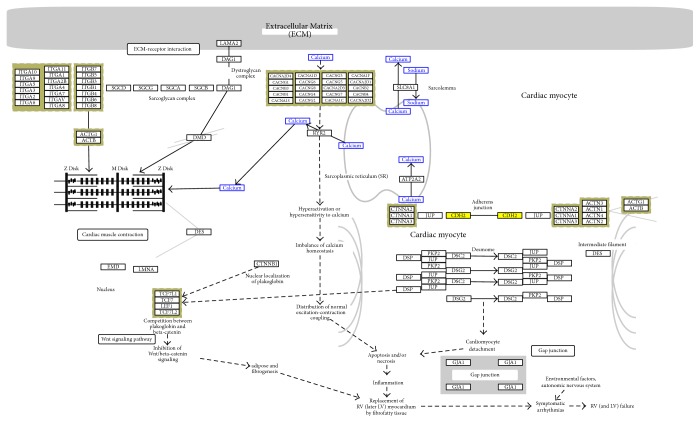
Arhythmogenic right ventricular cardiomyopathy (ARVC) KEGG pathway [18] highlighting CDH2 updated neural cadherin (NCAD) in yellow as an important biomarker while green colour represents the other genes present.

In pathway analysis, we focused on pathways of genes controlled by a maximum number of miRNAs that are marked as important biomarkers by Ingenuity Pathway analysis. One of the best examples is cell adhesion molecules pathway as shown in Figure 2 (adopted from http://www.genome.jp/kegg/pathway/hsa/hsa05412.html).

This pathway is controlled by most of miRNAs involved in our study. Among them, 8 miRNAs including miR-21-3p transcription regulation, miR-212-3p in kinase activity, miR-34a kinase activity, miR-302b kinase activity, miR-584b, miR-302b5p transporter, miR-338-3p transporter, and miR-491-3p as enzyme (see Supplementary Data) have already been confirmed and reported [22]. Few miRNA like miR-181, miR-345, and miR-10 which are controlling differentially expressed genes can be detected in precancerous stage [23]; hence, they can be important biomarkers to detect cancer at early stage. Several genes which are important biomarkers in our study have their reported significance like* DHPR*,* DSP*, and* CDH2*/*NCAD* [24, 25]. In a noteworthy study related to cell adhesion, it was reported that altered expression of cell adhesion molecules (CAMs) has been found in oral carcinoma, where loss of CAM expression is often seen in poorly differentiated lesions. However, upregulation of certain integrins, such as alpha(v)beta(6), has consistently been found in oral cancer suggesting that it may play an active role in disease progression [26]. Here CDH2 is an important biomarker of study that is highlighted as yellow in Figure 2 and Supplementary Figure 2 showing its involvement in oral cancer. N-Cadherin is an important member of cadherin family. It plays an important role in cellular differentiation. Alternation in function of N-cadherin leads to poor differentiation and hindrance in transendothelial migration leading towards cancer [27]. Transendothelial migration in a cancer cell is administrated by Src family kinases through N-cadherin. A cancerous cell can regulate the Src pathway of its neighbouring endothelial cells by phosphorylating the N- and E-cadherin. It causes the loss of connection between neighbouring endothelial cells by allowing the cancer cells to pass through [28, 29]. Significantly important CDH2 along with a particular set of miRNA can be potential diagnostic biomarkers set.

**Figure 3:**
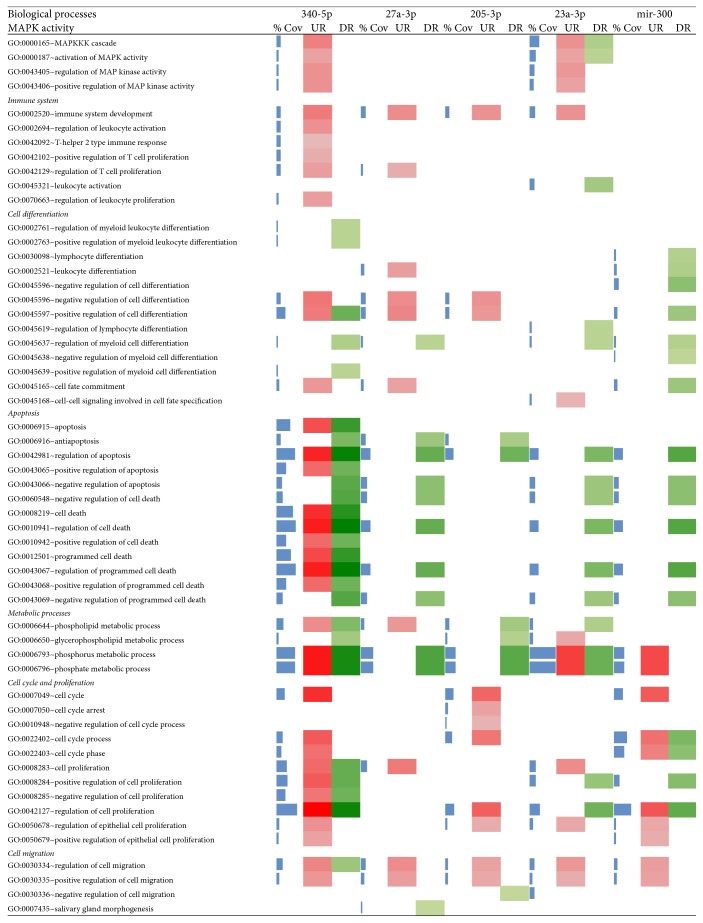
Biological processes of enrichment analysis by DAVID with miRNA target DEGs where colour intensities show the variation in expression (% Cov = blue, upregulated = red, and downregulated = green).

The functional analysis by DAVID uncovered extensive information on the role of miRNA in various biological processes, molecular functions, and construction of cellular compartments. These functions were shortlisted based on the criteria of relevance to cancer. Major categories of biological processes included MAPK activity, immune system, cell differentiation, apoptosis, metabolic processes, cell cycle, and proliferation as well as cell migration. Our results indicated that miR340-5p is the major player to control gene expression in biological processes. Biological processes related to MAPK pathway and immune systems were mostly upregulated whereas cell apoptosis, metabolic processes, and cell cycle, as well as cell proliferation, were almost equally affected by miR340-5p [30]. The miRNA deregulation in OSCC cell lines clearly reflected the involvement of miR340-5p in oral cancers. A trend of downregulation was observed in apoptosis by other 4 miRNAs: miR27a-3p, miR205-3p, miR23a-3p, and miR300 (Figure 3).

**Figure 4:**
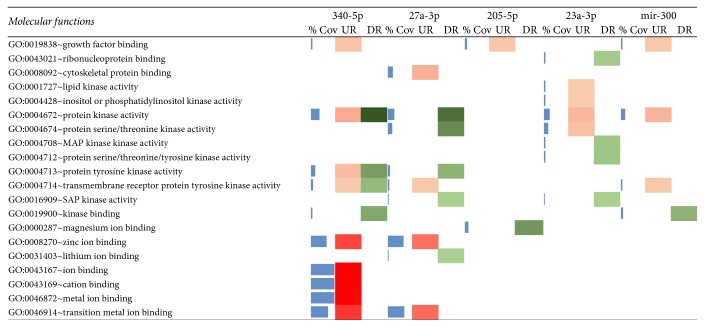
Molecular functions of enrichment analysis by DAVID with miRNA target DEGs where colour intensities show the variation in expression (% Cov = blue, upregulated = red, and downregulated = green).

The miR27a-3p is circulating not only extracellular miRNA but also a very significant biomarker in various other cancers like colorectal and breast cancers [1, 15, 31]. Hence, it could be a potential candidate for biomarkers in oral cancer too. The miR23a-3p along with two other miRNAs, that is, miR-193a-3p and miR-338-5p, has the synergistic ability for early detection of colorectal cancer [32]. Therefore, its significant involvement and presence with other four microRNAs can be considered as a significant set of biomarkers for early detection of oral cancers. Role of miR205 for the proliferation of oral cancers by regulation of LRP1 gene is already proven [33], which explains its downregulation in apoptosis. In molecular functions analysis, we observed a random pattern of gene expression with the highest contribution of miR340-5p. Almost all metal ion-binding systems were upregulated by miR340-5p, which suggests the high importance of this miRNA in molecular functions. Overall similar expression pattern was observed in target DEGs of each miRNA. The gene expression of the lumen was differentially affected by miRNA (Figure 4).

**Figure 5:**
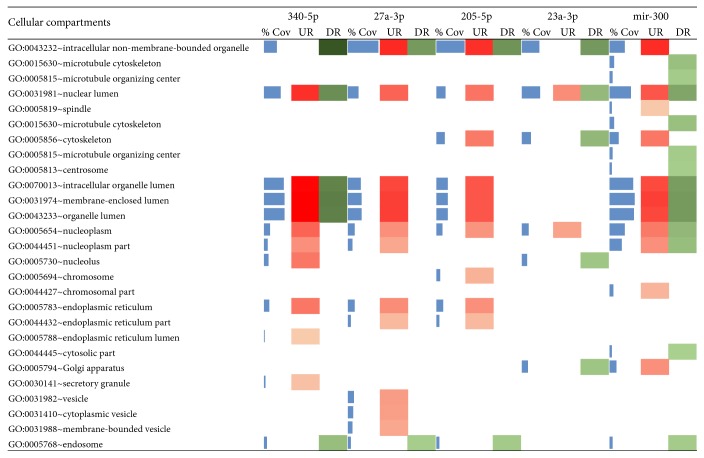
Cellular compartments of enrichment analysis by DAVID with miRNA target DEG where colour intensities show the variation in expression (% Cov = blue, upregulated = red, and downregulated = green).

Furthermore, the expression of genes in cellular compartments was also explored to study the role of miRNA cellular organelle structures as shown in Figure 5.

**Figure 6:**
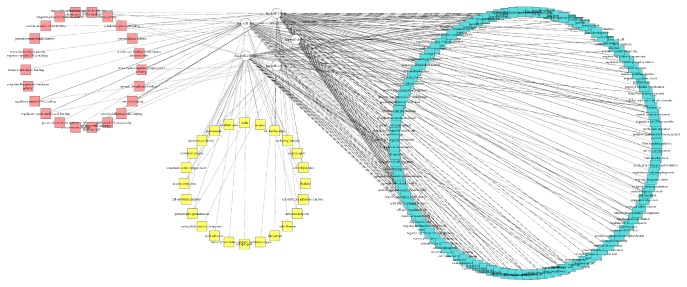
Network view of five miRNAs and their biological functions. The pink colour is representing molecular functions, while yellow is showing cellular compartments and greenish/bluish is expressing biological processes.

A similar study has been reported previously [34], where a very active involvement of lumen genes has been discussed. This is evident that miR340-5P which is controlling luminal genes can be a good candidate for biomarkers. The results from statistical analysis and functional enrichment analysis were presented in the form of interacting networks to assist their interpretation using software called Cytoscape (3.2.1) [35]. The network is shown in Figure 6 which is showing the interaction between miRNAs and their target genes, also mentioning gene's upregulated and downregulated behaviour.

The network is a clear depiction of the relationship between various biological processes, molecular functions, and cellular compartments and their corresponding controlling miRNAs. More than one miRNAs have been shown to be involved in controlling the same function while one function has been influenced by several different miRNAs. It is illustrating the connections between each of the miRNA and its functional role in major GO terms of biological processes, cellular compartments, or molecular functions as indicated by DAVID.

## 4. Conclusions

We have identified a list of differentially expressed genes that are being regulated by miRNA. These miRNAs are extracellular miRNAs and present in saliva. Hence, these miRNAs can be used as a significant set of the biomarkers in oral carcinoma. Furthermore, we have analyzed their pathways to verify their significance in other types of cancers that can be correlated in oral oncology. Functional enrichment analysis of the most significant miRNAs provided a solid in silico evidence of the importance of these miRNAs as biomarkers. We can use a proposed set of biomarkers in further scientific studies. These biomarkers can be validated using wet laboratory techniques. Any unique set of them can be used for immune-histochemistry techniques to identify the cancers at earliest possible stages.

## Figures and Tables

**Table 1:** Experimental techniques used for miRNA analysis.

Techniques	Number of experiments	Number of DEGs
Expression profiling by array	9	≈32,700
Genome variation profiling by array	2	≈86,150
Genome variation profiling by genome tiling array	2	≈9,730
Methylation profiling by array	2	≈12,880
Noncoding RNA profiling by array	1	≈63,500

## Additional Points

*Highlights*. (i) Possible targets of 318 extracellular salivary miRNAs were identified. (ii) A possible set of salivary biomarkers for early detection of oral cancers was proposed. (iii) Functional enrichment analysis of five most significant miRNAs in oral cancers was performed. (iv) The role of* CDH2* in relation to miRNA was identified. (v) The role of miR340-5p in metal ion-binding systems was highlighted.

## Supplementary Materials

Supplementary Materials Supplementary materials contain algorithms, tables, and figures. Two main algorithms are used in this paper. One is for retrieving common genes between two files. One file contains differentially expressed genes in oral cancer and another file contains overall target genes of miRNAs. Another algorithm was used for finding those genes that are the target of a maximum number of miRNAs. Table  1 contains selected experiments from the literature for the analysis in the current study. We used 16 experiments for this study and their details can be found in the table. Table  2 is composed of list of proposed biomarkers of oral cancer. It contains details about the list of genes with their location, controlling mRNA, and their type. Supplementary Figures  1(b–l) are showing the number of genes regulated against miRNA. They are representing their upregulation, downregulation, or nonregulation against particular miRNA. Supplementary Figure  2 is representing the cell adhesion molecules pathway in which CDH2 is represented as an important biomarker in this pathway. Algorithm  1: Retrieving common genes. Algorithm  2: Retrieving genes regulated by maximum number of miRNAs. Supplementary Table  1: selected experiments from the literature for the analysis in current study. Supplementary Table  2: list of proposed biomarkers of oral cancer. Supplementary Figure s 1(b–l): Blue, red, and green bars are showing the upregulation, nonregulation, and downregulation of genes, respectively. The miRNAs are shown on *x*-axis of graph while genes are represented on *y*-axis. Supplementary Figure  1(b): Number of genes regulated against miRNA. Supplementary Figure  1(c): Number of genes regulated against miRNA. Supplementary Figure  1(d): Number of genes regulated against miRNA. Supplementary Figure  1(e): Number of genes regulated against miRNA. Supplementary Figure  1(f): Number of genes regulated against miRNA. Supplementary Figure  1(g): Number of genes regulated against miRNA. Supplementary Figure  1(h): Genes regulated by miRNAs. Supplementary Figure  1(i): Number of genes regulated against miRNA. Supplementary Figure  1(j): Number of genes regulated by miRNAs. Supplementary Figure  1(k): Number of genes regulated against miRNA. Supplementary Figure  1(l): Number of genes regulated against miRNAs. Supplementary Figure  2. Cell adhesion molecules pathway in oral cancer patients. CDH2 is highlighted in yellow as important biomarker while green color represents the other genes present in this pathway (adopted from: http://www.genome.jp/kegg-bin/show_pathway?hsa04514).
