# Supplementary material for: Meta-Analysis of miRNAs and Their Involvement as Biomarkers in Oral Cancers
Source: Biomed Res Int. 2018 Jan 4;2018:8439820. doi: 10.1155/2018/8439820 (PMC5817319; doi:10.1155/2018/8439820)
Supplement: Supplementary Materials — contain algorithms, tables, and figures. Two main algorithms are used in this paper. One is for retrieving common genes between two files. One file contains differentially expressed genes in oral cancer and another file contains overall target genes of miRNAs. Another algorithm was used for finding those genes that are the target of a maximum number of miRNAs. Table 1 contains selected experiments from the literature for the analysis in the current study. We used 16 experiments for this study and their details can be found in the table. Table 2 is composed of list of proposed biomarkers of oral cancer. It contains details about the list of genes with their location, controlling mRNA, and their type. Supplementary Figures 1(b–l) are showing the number of genes regulated against miRNA. They are representing their upregulation, downregulation, or nonregulation against particular miRNA. Supplementary Figure 2 is representing the cell adhesion molecules pathway in which CDH2 is represented as an important biomarker in this pathway. Algorithm 1: Retrieving common genes. Algorithm 2: Retrieving genes regulated by maximum number of miRNAs. Supplementary Table 1: selected experiments from the literature for the analysis in current study. Supplementary Table 2: list of proposed biomarkers of oral cancer. Supplementary Figure s 1(b–l): Blue, red, and green bars are showing the upregulation, nonregulation, and downregulation of genes, respectively. The miRNAs are shown on x-axis of graph while genes are represented on y-axis. Supplementary Figure 1(b): Number of genes regulated against miRNA. Supplementary Figure 1(c): Number of genes regulated against miRNA. Supplementary Figure 1(d): Number of genes regulated against miRNA. Supplementary Figure 1(e): Number of genes regulated against miRNA. Supplementary Figure 1(f): Number of genes regulated against miRNA. Supplementary Figure 1(g): Number of genes regulated against miRNA. Supplementary Figure 1(h): G [file 8439820.f1.doc]

**Supplementary material:**

# Algorithms used:

Two main algorithms are used in this paper. One is for retrieving common genes between two files. One file contains differentially expressed genes in oral cancer and other file contains overall target genes of miRNAs. Other algorithm is used for finding those genes that are target of maximum number of miRNAs.

## Algorithm 1: Retrieving common genes

## Algorithm 2: Retrieving genes regulated by maximum number of miRNAs

# Tables:

## Supplementary Table 1: Selected experiments from the literature for the analysis in current study.

| **Sr. No** | **Experiment** | **Study type** | **References** |
| --- | --- | --- | --- |
| 1. | Genome wide expression profiling of anterior tongue cancer with no history of tobacco and alcohol use. | Expression profiling by array. |  |
| 2. | Squamous cell carcinoma of the tongue: tumors and histologically normal surgical margins | Expression profiling by array. | - Reis et al., 2011 |
| 3. | Oral squamous cell carcinoma-derived cell line | Expression profiling by array. | Yap et al., 2009 |
| 4. | Genome wide expression profiling of anterior tongue cancer with no history of tobacco and alcohol use | Expression profiling by array. |  |
| 5. | SBV - Gene Expression Profiles of Lung Cancer Tumors– Adenocarcinomas and Squamou Cell Carcinomas. | Expression profiling by array. | Tarca et al., 2013 |
| 6. | Gene expression profiling of oral squamous cell carcinoma (OSCC). | Expression profiling by array. | Sheu et al., 2014 |
| 7. | Squamous cell carcinoma of the oral cavity with lymph node metastasis. | Expression profiling by array. | O'Donnell et al., 2005 |
| 8. | Global analysis of DNA methylation changes during progression of oral tumorigenesis. | Expression profiling by array. | Towle et al.,2013 |
| 9. | Integrative genomic characterization of oral squamous cell carcinoma identifies frequent somatic drivers (methylation). | Expression profiling by array. | Pickering et al.,2013 |
| 10. | Global analysis of DNA methylation changes during progression of oral tumorigenesis | Methylation profiling  by array. | Towle et al., 2013 |
| 11. | NID2 and HOXA9 promoter hypermethylation as biomarkers to prevent and detect Oral Cavity Squamous Cell Carcinoma. | Methylation profiling  by array. | Guerrero-Preston et al., 2011 |
| 12. | Chromosomal aberrations and aneuploidy in oral potentially malignant lesions: distinctive features for tongue. | Genome variation profiling by genome tiling array. | Castagnola et al., 2011 |
| 13. | Array CGH analysis of Oral Squamous Cell Carcinoma | Genome variation profiling by genome tiling array. | Ambatipudi et al., 2011 |
| 14. | Analysis of Molecular Alterations in Oral Cell Lines | Genome variation profiling by array. | Dickman et al., 2014 |
| 15. | Genomic profiling of oral squamous cell carcinoma by array based comparative genomic hybridization | Genome variation profiling by array. | Yoshioka et al., 2013 |
| 16. | MiRNA expression profile in Head and Neck Cancer: HOX-cluster embedded miRNA-196 and miRNA-10b deregulation is implicated in cell proliferation | Non-coding RNA profiling by array. | Severino et al., 2013 |

## Supplementary Table 2: List of proposed biomarkers of oral cancer.

| Symbol | Entrez Gene Name | Location | No. of miRNA regulates gene | miRNAs | Type(s) |
| --- | --- | --- | --- | --- | --- |
| ABCF3 | ATP-binding cassette, sub-family F (GCN20), member 3 | Other | 4 | miR-15a-5p, miR-455-3p, miR-503, miR-15b-5p | Transporter |
| ABT1 | activator of basal transcription 1 | Nucleus | 12 | miR-98, miR-154-3p, miR-378g, miR-644b-5p, miR-7e-5p, let-7a-5p, let-7b-5p, let-7c, let-7d-5p, let-7f-5p, let-7i-5p, let-7g-5p | transcription regulator |
| AHRR | aryl-hydrocarbon receptor repressor | Nucleus | 14 | miR-92b-3p, miR-124-3p, miR-140-3p, miR-155-5p, miR-195-3p, miR-200b-3p, miR-200c-3p, miR-296-3p, miR-331-5p, miR-374c-5p, miR-382-5p, miR-512-3p, miR-524-5p, miR-601 | Other |
| ALDH2 | aldehyde dehydrogenase 2 family (mitochondrial) | Cytoplasm | 10 | miR-15a-5p, miR-92a-2-5p, miR-193b-3p, miR-195-5p, miR-302a-5p, miR-328, miR-491-5p, miR-15b-5p, miR-16-5p, miR-22-5p | Enzyme |
| ANXA2 | annexin A2 | Plasma Membrane | 5 | miR-130b-5p, miR-206, miR-425-5p, miR-659-5p, miR-1 | Other |
| ARF6 | ADP-ribosylation factor 6 | Plasma Membrane | 3 | miR-28-3p, miR-378g, miR-524-5p | Transporter |
| ARFGAP1 | ADP-ribosylation factor GTPase activating protein 1 | Cytoplasm | 1 | miR-338-3p | Transporter |
| ARPC2 | actin related protein 2/3 complex, subunit 2, 34kDa | Cytoplasm | 7 | miR-29a-3p, miR-29b-3p, miR-29c-3p, miR-146b-3p, miR-200a-3p, miR-200b-3p, miR-200c-3p | Other |
| CAPZA2 | capping protein (actin filament) muscle Z-line, alpha 2 | Cytoplasm | 26 | miR-15a-5p, miR-23a-3p, miR-34a-3p, miR-100-3p, miR-103a-3p, miR-107, miR-195-5p, miR-197-3p, miR-200b-3p, miR-200c-3p, miR-205-3p, miR-205r-3p, miR-211-3p, miR-300, miR-338-5p, miR-371b-5p, miR-373-5p, miR-374a-5p, miR-374b-5p, miR-374c-5p, miR-503, miR-15b-5p, miR-16-5p, miR-23b-3p, let-7a-3p, let-7b-3p | Other |
| CD163L1 | CD163 molecule-like 1 | Plasma Membrane | 3 | miR-140-5p, miR-148a-3p, miR-645 | transmembrane receptor |
| CDH2 | cadherin 2, type 1, N-cadherin (neuronal) | Plasma Membrane | 34 | miR-26-5p, miR-26a-5p, miR-106-3p, miR-124-3p, miR-181b-3p, miR-194-5p, miR-199a-5p, miR-199b-5p, miR-320a, miR-320b, miR-320c, miR-320d, miR-320e, miR-367-5p, miR-374c-5p, miR-484, miR-499c-3p, miR-524-5p, miR-587, miR-589-3p, miR-649, miR-9-3p, let-7a-2-3p, let-7g-3p | Other |
| CDK7 | cyclin-dependent kinase 7 | Nucleus | 4 | miR-382-5p, miR-501-5p, miR-587, miR-7-5p | Kinase |
| CENPBD1 | CENPB DNA-binding domains containing 1 | Other | 15 | miR-24-3p, miR-27a-5p, miR-95, miR-103a-3p, miR-106-3p, miR-107, miR-149-5p, miR-195-5p, miR-204-3p, miR-215, miR-373-5p, miR-374a-3p, miR-378g, let-7a-2-3p, let-7g-3p | Other |
| CENPN | centromere protein N | Nucleus | 6 | miR-19a-3p, miR-19b-3p, miR-205-3p, miR-205r-3p, miR-223-3p, miR-455-5p | Other |
| CHP1 | calcineurin-like EF-hand protein 1 | Cytoplasm | 21 | miR-26-3p, miR-30b-3p, miR-30c-1, miR-30c-2, miR-34b-5p, miR-124-3p, miR-140-5p, miR-146b-3p, miR-200a-3p, miR-214-3p, miR-302a-5p, miR-337-3p, miR-365a-3p, miR-365b-3p, miR-367-3p, miR-499a-3p, miR-499c-5p, miR-644a, miR-7-5p, miR-22-5p, let-7a-3p | Transporter |
| CTSD | cathepsin D | Cytoplasm | 4 | miR-92a-2-5p, miR-128, miR-130b-3p, miR-342-3p | Peptidase |
| DENND4C | DENN/MADD domain containing 4C | Other | 26 | miR-19a-3p, miR-19b-3p, miR-30a-5p, miR-30b-5p, miR-30d-5p, miR-30e-5p, miR-34b-5p, miR-124-3p, miR-125b-2-3p, miR-148a-3p, miR-194-5p, miR-203, miR-205-3p, miR-205r-3p, miR-300, miR-302a-5p, miR-338-5p, miR-345-3p, miR-371a-5p, miR-374a-5p, miR-499a-5p, miR-499c-5p, miR-520c-3p, miR-520e, miR-524-5p, miR-644b-3p | Other |
| DPH5 | diphthamide biosynthesis 5 | Other | 1 | miR-92a-2-5p | Enzyme |
| DYRK2 | dual-specificity tyrosine-(Y)-phosphorylation regulated kinase 2 | Cytoplasm | 69 | miR-19a-3p, miR-19b-3p, miR-24-3p, miR-25-3p, miR-29a-3p, miR-29b-3p, miR-29c-3p, miR-34a-5p, miR-92b-3p, miR-98, miR-101-3p, miR-103a-3p, miR-106-3p, miR-107, miR-124-3p, miR-125a-5p, miR-125b-5p, miR-126-5p, miR-127-5p, miR-132-3p, miR-137, miR-142-5p, miR-148a-3p, miR-155-5p, miR-200a-3p, miR-200b-3p, miR-200c-3p, miR-212-3p, miR-224-3p, miR-224-5p, miR-302a-3p, miR-302a-5p, miR-302b-3p, miR-302b-5p, miR-302c-3p, miR-302c-5p, miR-302d-3p, miR-302d-5p, miR-320a, miR-320b, miR-320c, miR-320d, miR-338-5p, miR-340-5p, miR-365a-3p, miR-365b-3p, miR-367-3p, miR-371a-5p, miR-372, miR-373-3p, miR-374a-5p, miR-499a-5p, miR-512-3p, miR-520c-3p, miR-520e, miR-524-5p, miR-589-3p, miR-644b-3p, miR-649, miR-659-3p, miR-7e-5p, miR-9-3p, miR-20a-5p, let-7a-5p, let-7b-5p, let-7c, let-7f-5p, let-7i-5p, let-7g-5p | Kinase |
| EHD3 | EH-domain containing 3 | Cytoplasm | 9 | miR-133a, miR-133b, miR-147a, miR-204-3p, miR-205-3p, miR-205r-3p, miR-372, miR-587, miR-20a-5p | Other |
| ERCC3 | excision repair cross-complementation group 3 | Nucleus | 20 | miR-10b-5p, miR-30a-5p, miR-30b-5p, miR-30d-5p, miR-30e-5p, miR-34b-5p, miR-193a-3p, miR-193b-3p, miR-212-5p, miR-367-5p, miR-371a-3p, miR-378b, miR-378d, miR-378e, miR-378f, miR-378g, miR-378h, miR-499c-5p, miR-587, miR-589-3p | Enzyme |
| ETFA | electron-transfer-flavoprotein, alpha polypeptide | Cytoplasm | 15 | miR-148a-5p, miR-203,miR-205-3p, miR-205r-3p, miR-219-5p, miR-302b-5p, miR-302d-5p, miR-320a, miR-320b, miR-320c, miR-320d, miR-337-3p, miR-374a-5p, miR-374b-5p, miR-520c-5p | Transporter |
| ETV6 | ets variant 6 | Nucleus | 6 | miR-18a-5p, miR-18b-5p, miR-130b-5p, miR-378g, miR-587, miR-21 | transcription regulator |
| EXT1 | exostosin glycosyltransferase 1 | Cytoplasm | 65 | miR-15a-5p, miR-23a-3p, miR-24-1-5p, miR-24-2-5p, miR-26-3p, miR-26-5p, miR-26a-5p, miR-29a-3p, miR-29b-3p, miR-29c-3p, miR-30d-3p, miR-30e-3p, miR-96-5p, miR-103b, miR-125b-2-3p, miR-134, miR-142-5p, miR-145-5p, miR-148a-5p, miR-149-5p, miR-154-3p, miR-155-5p, miR-181b-5p, miR-181d, miR-195-5p, miR-196a-3p, miR-197-5p, miR-200b-3p, miR-200c-3p, miR-204-3p, miR-205-3p, miR-205r-3p, miR-212-5p, miR-224-5p, miR-300, miR-302a-3p, miR-302b-3p, miR-302c-3p, miR-302d-3p, miR-342-3p, miR-371a-5p, miR-371b-5p, miR-372, miR-373-3p, miR-373-5p, miR-374c-5p, miR-375, miR-382-3p, miR-503, miR-520c-3p, miR-520e, miR-524-5p, miR-587, miR-589-5p, miR-644b-3p, miR-649, miR-15b-5p, miR-16-5p, miR-20a-5p, miR-22-5p, miR-23b-3p, let-7a-2-3p, let-7a-3p, let-7b-3p, let-7g-3p | Enzyme |
| FADS2 | Fatty acid desaturase 2 | Plasma Membrane | 4 | miR-92a-2-5p, miR-149-3p, miR-491-5p, miR-589-3p | Enzyme |

# Figures

**Supplementary Figure 1(b-l)**: Blue, red and green bars are showing the up-regulation, non-regulation and down-regulation of genes, respectively. The miRNAs are shown on x-axis of graph while genes are represented on y-axis.


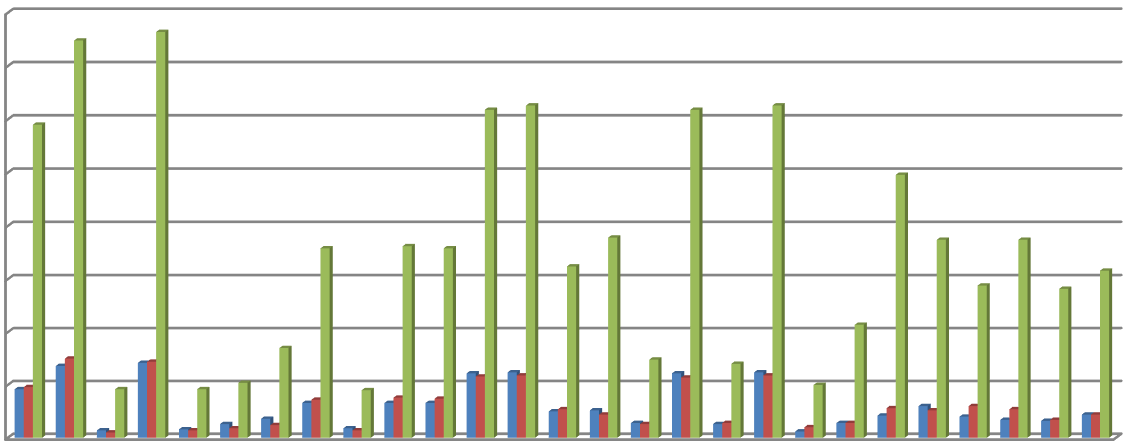


0

50

100

150

200

250

300

350

400

mir-26a-5p

mir-27a-3p

mir-27a-5p

mir-27b-3p

mir-27b-5p

mir-28-3p

mir-28-5p

mir-29a-3p

mir-29a-5p

mir-29b-3p

mir-29c-3p

mir-30a-5p

mir-30b-5p

mir-30c-1

mir-30c-2

mir-30d-3p

mir-30d-5p

mir-30e-3p

mir-30e-5p

mir-31-3p

mir-31-5p

mir-34a-3p

mir-34a-5p

mir-34b-3p

mir-34b-5p

mir-34c-3p

mir-34c-5p

UP

NON

DOWN

**Supplementary Figure 1 (b): Number of genes regulated against miRNA.**


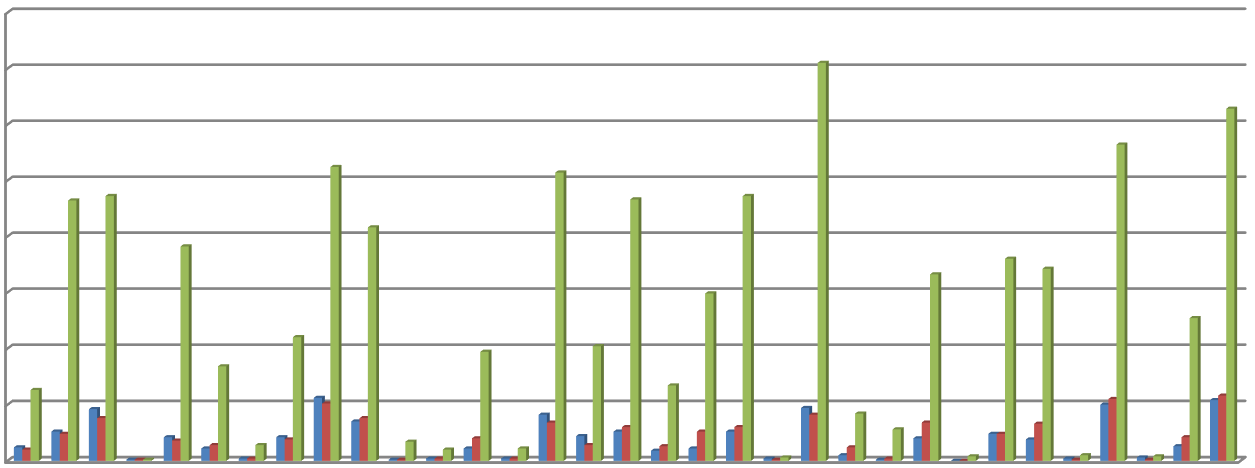


0

50

100

150

200

250

300

350

400

mir-92a-1-5p

mir-92a-2-5p

mir-92b-3p

mir-92b-5p

mir-93-3p

mir-93-5p

mir-95

mir-96-3p

mir-96-5p

mir-98

mir-99a-3p

mir-99a-5p

mir-100-3p

mir-100-5p

mir-101-3p

mir-101-5p

mir-103a-3p

mir-103b

mir-106-3p

mir-107

mir-122-3p

mir-124-3p

mir-124-5p

mir-125a-3p

mir-125a-5p

mir-125b-1-3p

mir-125b-2-3p

mir-125b-5p

mir-126-3p

mir-126-5p

mir-127-3p

mir-127-5p

mir-128

UP

NON

DOWN

**Supplementary Figure 1 (c): Number of genes regulated against miRNA**


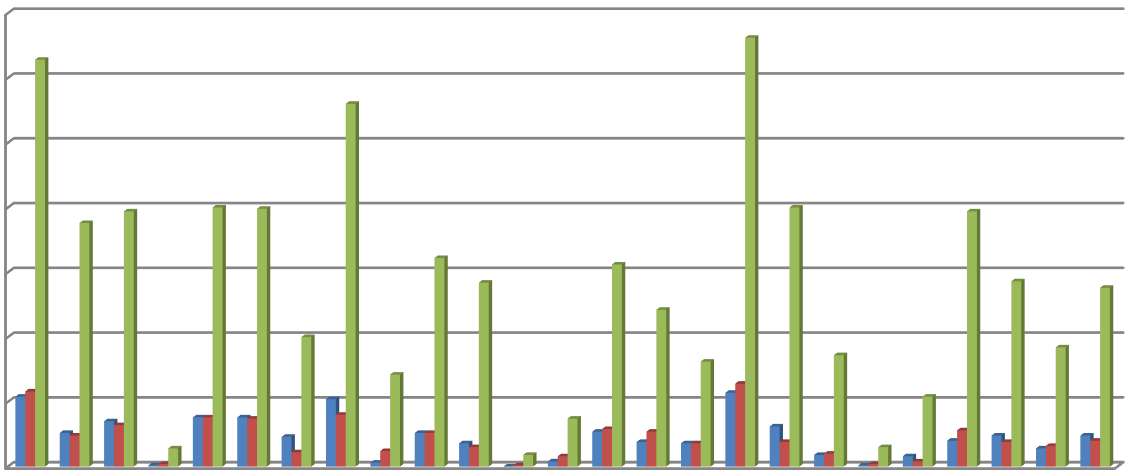


0

50

100

150

200

250

300

350

mir-130b-3p

mir-130b-5p

mir-132-3p

mir-132-5p

mir-133a

mir-133b

mir-134

mir-137

mir-138-1-3p

mir-138-2-3p

mir-138-5p

mir-139-3p

mir-139-5p

mir-140-3p

mir-140-5p

mir-142-3p

mir-142-5p

mir-143-3p

mir-143-5p

mir-145-3p

mir-145-5p

mir-146a-3p

mir-146a-5p

mir-146b-3p

mir-146b-5p

UP

NON

DOWN

**Supplementary Figure 1(d): Number of genes regulated against miRNA**

**
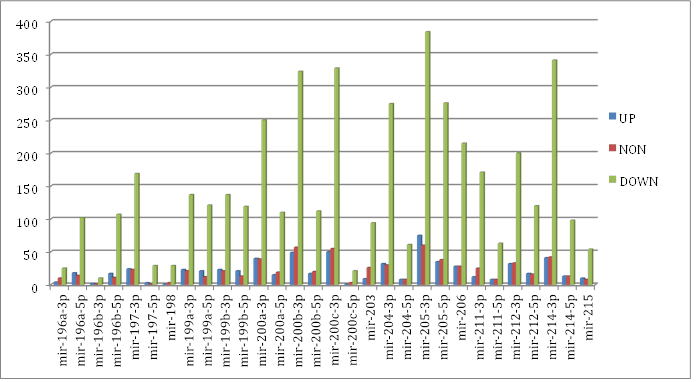
**

**Supplementary Figure 1 (e): Number of genes regulated against miRNA**


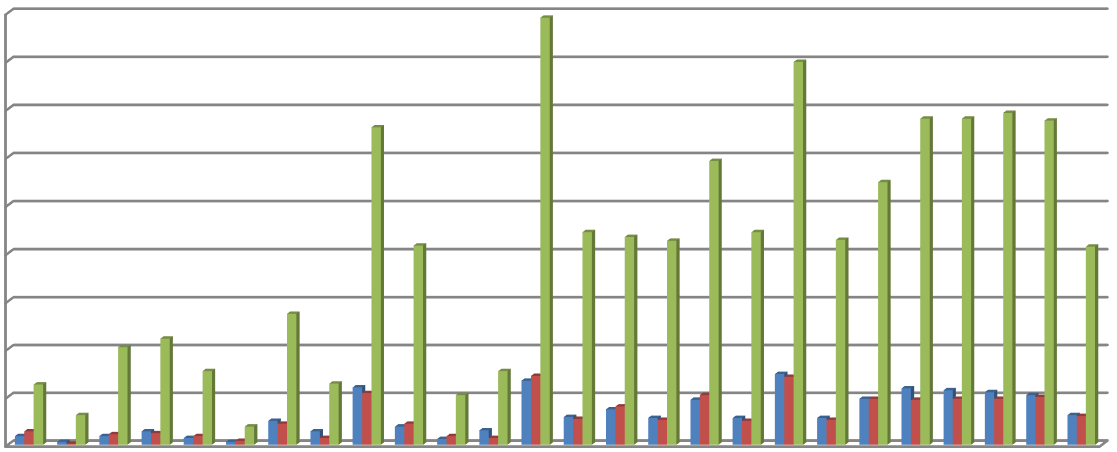


0

50

100

150

200

250

300

350

400

450

mir-218-5p

mir-219-5p

mir-221-3p

mir-221-5p

mir-222-3p

mir-222-5p

mir-223-3p

mir-223-5p

mir-224-3p

mir-224-5p

mir-296-3p

mir-296-5p

mir-300

mir-302a-3p

mir-302a-5p

mir-302b-3p

mir-302b-5p

mir-302c-3p

mir-302c-5p

mir-302d-3p

mir-302d-5p

mir-320a

mir-320b

mir-320c

mir-320d

mir-320e

UP

NON

DOWN

**Supplementary Figure 1 (f) Number of genes regulated against miRNA**


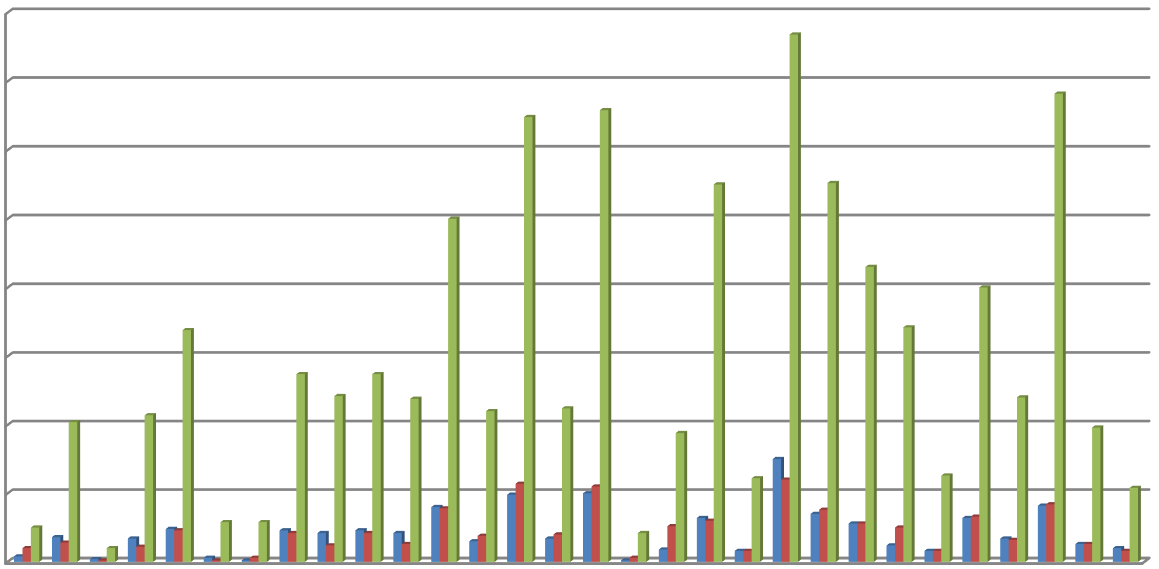


0

50

100

150

200

250

300

350

400

mir-196a-3p

mir-196a-5p

mir-196b-3p

mir-196b-5p

mir-197-3p

mir-197-5p

mir-198

mir-199a-3p

mir-199a-5p

mir-199b-3p

mir-199b-5p

mir-200a-3p

mir-200a-5p

mir-200b-3p

mir-200b-5p

mir-200c-3p

mir-200c-5p

mir-203

mir-204-3p

mir-204-5p

mir-205-3p

mir-205-5p

mir-206

mir-211-3p

mir-211-5p

mir-212-3p

mir-212-5p

mir-214-3p

mir-214-5p

mir-215

UP

NON

DOWN

**Supplementary Figure 1 (g): Number of genes regulated against miRNA**


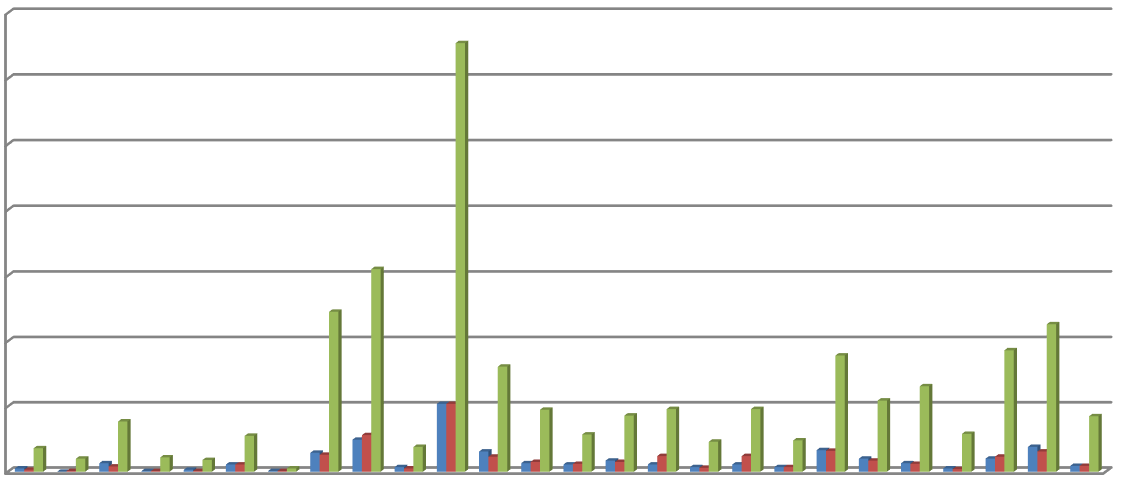


0

100

200

300

400

500

600

700

mir-324-5p

mir-325

mir-328

mir-331-3p

mir-331-5p

mir-337-3p

mir-337-5p

mir-338-3p

mir-338-5p

mir-340-3p

mir-340-5p

mir-342-3p

mir-345-3p

mir-345-5p

mir-346

mir-365a-3p

mir-365a-5p

mir-365b-3p

mir-365b-5p

mir-367-3p

mir-367-5p

mir-370

mir-371a-3p

mir-371a-5p

mir-371b-5p

mir-371b-3p

UP

NON

DOWN

**Supplementary Figure 1 (h): Genes regulated by miRNAs**


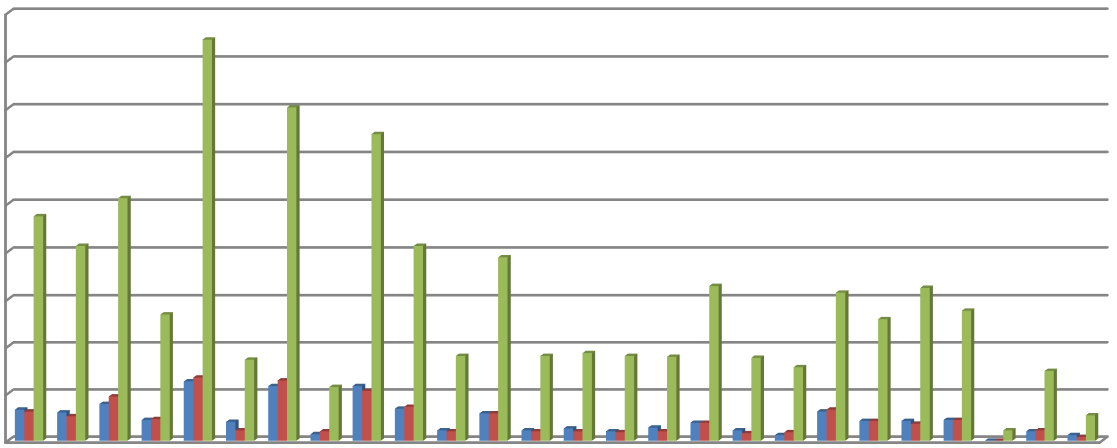


0

50

100

150

200

250

300

350

400

450

mir-372

mir-373-3p

mir-373-5p

mir-374a-3p

mir-374a-5p

mir-374b-3p

mir-374b-5p

mir-374c-3p

mir-374c-5p

mir-375

mir-378a-3p

mir-378a-5p

mir-378b

mir-378c

mir-378d

mir-378f

mir-378g

mir-378h

mir-378i

mir-382-3p

mir-382-5p

mir-423-5p

mir-425-5p

mir-451a

mir-455-3p

mir-455-5p

UP

NON

DOWN

**Supplementary Figure 1 (i): Number of genes regulated against miRNA**


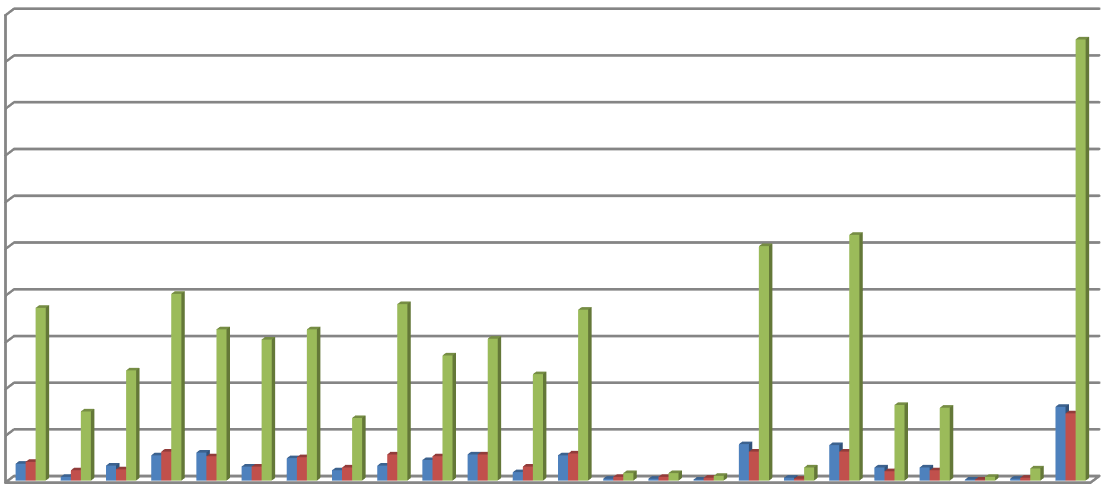


0

50

100

150

200

250

300

350

400

450

500

mir-484

mir-491-3p

mir-491-5p

mir-499a-3p

mir-499a-5p

mir-499b-3p

mir-499b-5p

mir-499c-3p

mir-499c-5p

mir-501-3p

mir-501-5p

mir-503

mir-512-3p

mir-517b-3p

mir-517c-3p

mir-518b

mir-520c-3p

mir-520c-5p

mir-520e

mir-520g

mir-520h

mir-521

mir-524-3p

mir-524-5p

UP

NON

DOWN

**Supplementary Figure 1 (j): Number of genes regulated by miRNAs**


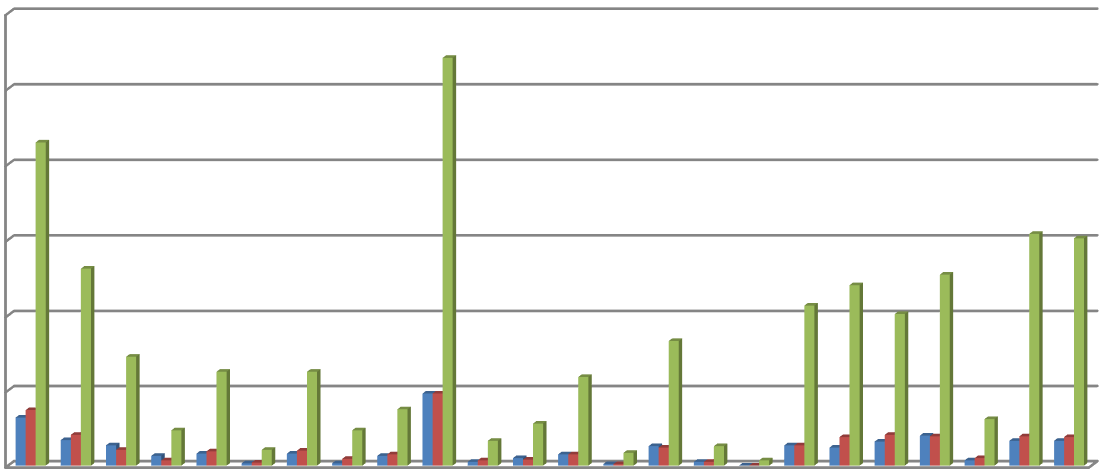


0

100

200

300

400

500

600

mir-587

mir-589-3p

mir-589-5p

mir-601

mir-642a-3p

mir-642a-5p

mir-642b-3p

mir-642b-5p

mir-644a

mir-644b-3p

mir-644b-5p

mir-645

mir-649

mir-658

mir-659-3p

mir-659-5p

mir-425-3p

mir-1

mir-7-5p

mir-7e-5p

mir-9-3p

mir-15b-3p

mir-15b-5p

mir-16-5p

UP

NON

DOWN

**Supplementary Figure 1 (k): Number of genes regulated against miRNA**


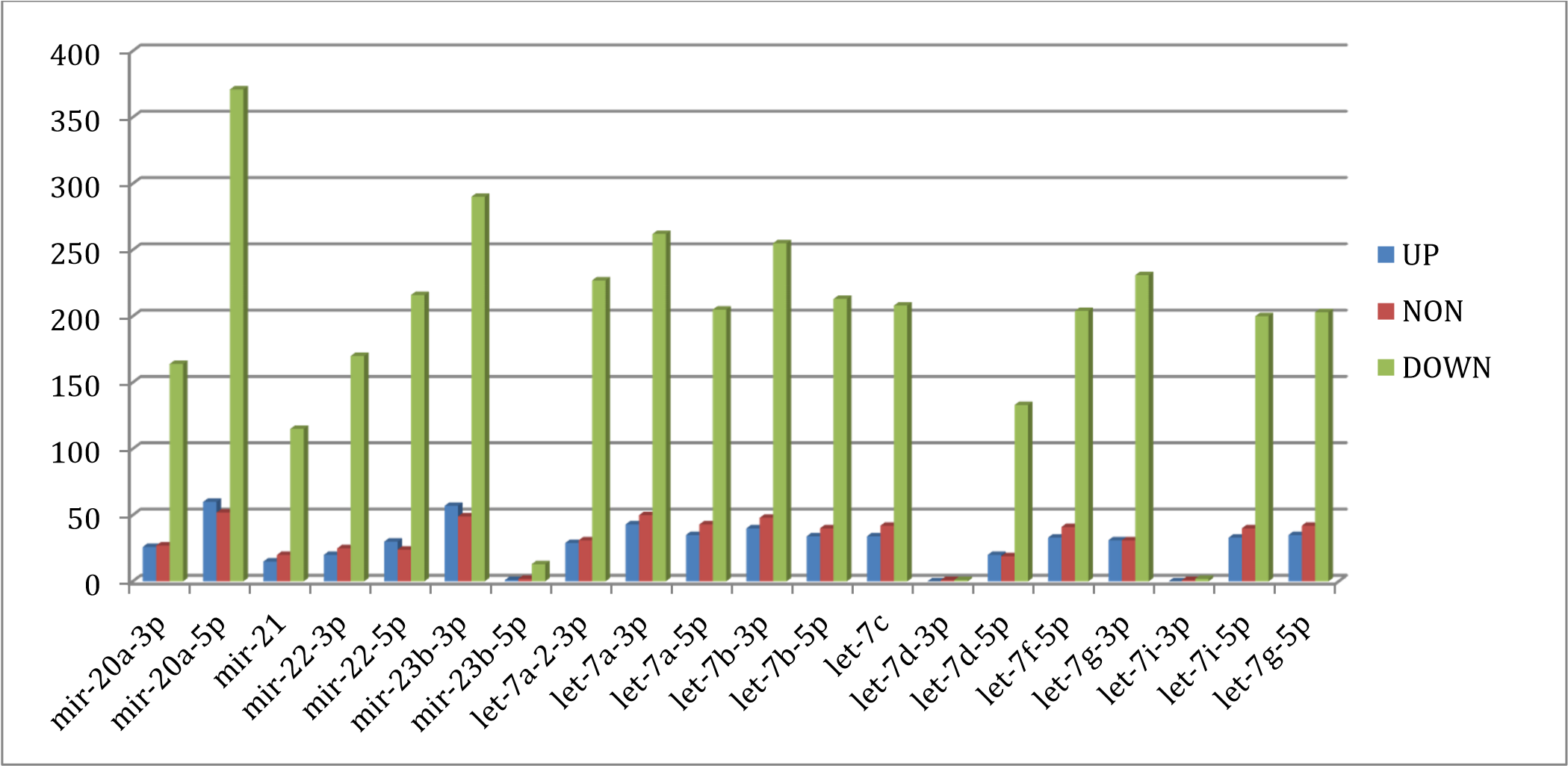


**Supplementary Figure 1 (l): Number of genes regulated against miRNAs**


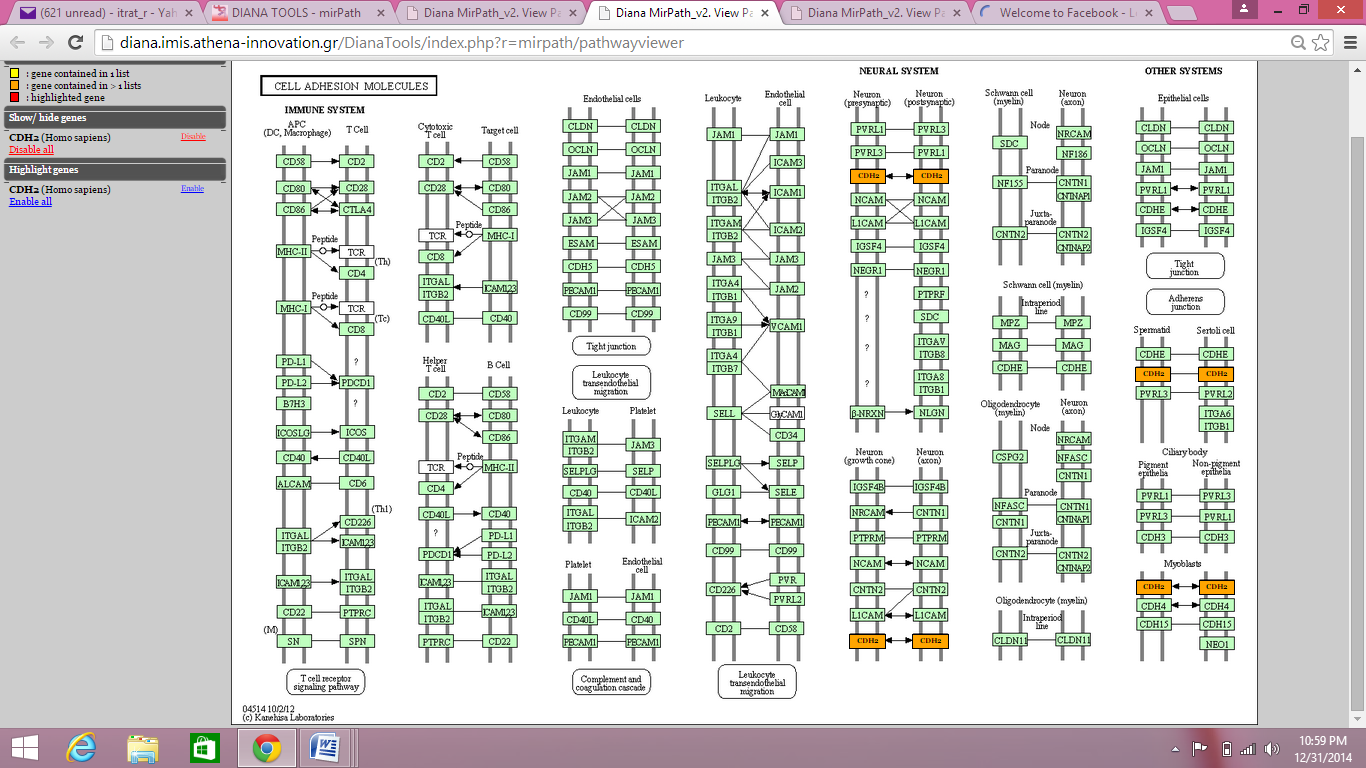


Supplementary Figure 2. Cell Adhesion Molecules Pathway in oral cancer patients CDH2 is highlighted in yellow as important biomarker while green color represents the other genes present in this pathway(Adopted from: http://www.genome.jp/kegg-bin/show_pathway?hsa04514).
